# Personal

**Published:** 1910-01

**Authors:** 


### PERSONAL.

Dr. George A. Sloan, of Buffalo, has been appointed attending neurologist to the Erie County Hospital, vice William C. Krauss, deceased.

Dr. Nelson W. Wilson, of Buffalo, has been appointed Press Secretary to the Board of Trustees of the Buffalo General Hospital. This is a newly-created office, Dr. Wilson being the first incumbent. No one who knows Dr. Wilson will challenge his fitness for the place. The press notices of the general hospital in future will be prepared with accuracy and care.

Dr. Albert H. Garvin has been appointed superintendent of the New York State Hospital for Incipient Tuberculosis at Raybrook by the trustees of that institution. Dr. Garvin who was first physician at the hospital, has been the acting superintendent for the last two and a half years.

Dr. Joseph H. Carr, Dr. Thew Wright and Dr. Francis M. McGuire have been appointed attending surgeons at the Emergency Hospital to fill vacancies caused by the resignations of Dr. Joseph Burke, Dr. Vertner Kenerson and Dr. Edward M. Dooley, all of Buffalo.

Dr. Charles N. Palmer, of Lockport, was elected president of the Academy of Medicine of that city at its recent annual meeting. Dr. Palmer is one of the best known physicians in western New York, and the Lockport Academy of Medicine has done itself honor in choosing him for president.

